# Machine‐Learning‐Assisted Autonomous Humidity Management System Based on Solar‐Regenerated Super Hygroscopic Complex

**DOI:** 10.1002/advs.202003939

**Published:** 2021-02-01

**Authors:** Xueping Zhang, Jiachen Yang, Hao Qu, Zhi Gen Yu, Dilip Krishna Nandakumar, Yaoxin Zhang, Swee Ching Tan

**Affiliations:** ^1^ Department of Materials Science and Engineering National University of Singapore Singapore 117574 Singapore; ^2^ Institute of High Performance Computing Singapore 138632 Singapore

**Keywords:** cobalt complexes, humidity management, machine learning, solar regeneration, water harvesting

## Abstract

High levels of humidity can induce thermal discomfort and consequent health disorders. However, proper utilization of this astounding resource as a freshwater source can aid in alleviating water scarcity. Herein, a low‐energy and highly efficient humidity control system is reported comprising of an in‐house developed desiccant dehumidifier and hygrometer (sensor), with an autonomous operation capability that can realize simultaneous dehumidification and freshwater production. The high efficiency and energy saving mainly come from the deployed super hygroscopic complex (SHC), which exhibits high water uptake (4.64 g g^−1^) and facile regeneration. Machine‐learning‐assisted in‐house developed low cost and high precision hygrometers enable the autonomous operation of the humidity management system. The dehumidifier can reduce the relative humidity (RH) of a confined room from 75% to 60% in 15 minutes with energy consumption of 0.05 kWh, saving more than 60% of energy compared with the commercial desiccant dehumidifiers, and harvest 10 L of atmospheric water in 24 h. Moreover, the reduction in RH from 80% to 60% at 32 °C results in the reduction of apparent temperature by about 7 °C, thus effectively improving the thermal comfort of the inhabitants.

## Graphical abstract

A highly efficient, low‐energy, and autonomous desiccant‐based humidity control system comprising of an in‐house fabricated desiccant dehumidifier and hygrometer (sensor) is developed that can realize simultaneous dehumidification and freshwater production.

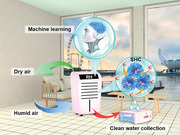

## 1 Introduction

The earth's atmosphere houses about 13 000 trillion litres of water at any given time.^[^ ^1^, ^2^ ^]^ Harvesting this water has become one of the hot research topics in recent years,^[^ ^3^, ^4^, ^5^, ^6^, ^7^, ^37^, ^39^ ^]^ since freshwater scarcity is one of the greatest challenges worldwide. On the other hand, in some humid regions, atmospheric water is usually considered as a redundant resource. Highly humid environment may induce the growth of mould, fungi, bacteria, and viruses, giving rise to respiratory discomfort, allergies, and infectious diseases.^[^ ^8^ ^]^ Recent studies have also corroborated the negative effect of high heat and humidity on an individual's performance over a wide range of activities in addition to direct health outcomes.^[^ ^9^ ^]^ Universally accepted appropriate relative humidity (RH) levels for a healthy and comfortable indoor environment range from 40% to 60%.^[^ ^10^, ^11^ ^]^ Higher the RH, higher is the “feel‐like” temperature and higher the thermal discomfort. Rather than looking at atmospheric moisture as a redundant resource, that requires additional energy input to maintain at comfortable levels, proper utilization of this astounding resource as a freshwater source may also aid in alleviating water scarcity that is prevalent throughout the globe.

Conventional cooling‐based dehumidifying systems dehumidify air by cooling it below the dew point temperature to remove moisture via condensation, and then reheating it to the desired temperature. These systems have inherent drawbacks associated with their operations which include their high energy consumption^[^ ^12^, ^13^ ^]^ and the use of refrigerants that may contribute to global warming.^[^ ^14^ ^]^ As an alternative, desiccant‐based dehumidification systems have garnered much attention in recent years owing to their energy efficiency, environmental friendliness, and a sustainable manufacturing process.^[^ ^15^, ^16^, ^17^ ^]^ However, current desiccant‐based dehumidification systems loaded with commercial desiccants such as silica gel and zeolites, have a higher energy consumption owing to the high regeneration temperatures of the desiccants used.^[^ ^18^, ^19^, ^20^, ^21^ ^]^

To bring down the RH to a comfortable level and realize the atmospheric water harvesting at the same time, we reported a highly efficient and low‐energy desiccant dehumidifier that was developed via 3D printing. The high efficiency and energy saving of the dehumidifier mainly comes from the desiccant that we have synthesized—a cobalt‐based super hygroscopic complex (SHC), which has superior moisture adsorption properties and low regeneration temperature (as low as 60 °C), thereby enabling facile regeneration, either through natural sunlight or low‐grade heat sources. This desiccant dehumidifier can continuously dehumidify the indoor environment and produce freshwater concurrently. In an attempt to make the operation of this dehumidifier autonomous, we have developed a machine learning‐assisted hygrometer (RH sensor) and integrated it with the dehumidifier to develop a humidity management system. This ensures minimal human intervention and additional energy savings. The hygrometer (sensor) was fabricated by using the changes in electrical resistance of the SHC upon moisture sorption. The sensory funtions were accurate, reliable and easy to manufacture and deploy.

## 2 Results and Discussion

### 2.1 Synthesis and Characterizations of SHC

**Figure 1:**
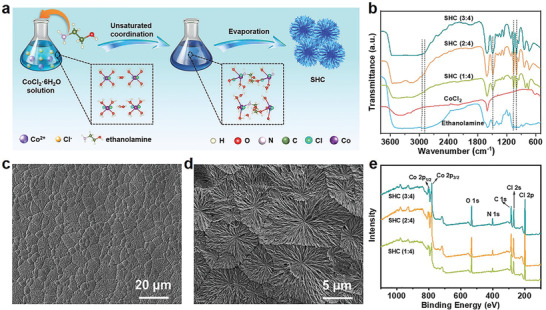
Synthesis and characterizations of SHC. a) The fabrication process of SHC. b) The FTIR spectra of ethanolamine, CoCl_2,_ and SHC prepared with different molar ratios of ethanolamine to CoCl_2_. c,d) SEM images of SHC. e) The XPS survey spectra of SHC prepared with different molar ratios of ethanolamine to CoCl_2_.

As illustrated in **Figure** **1**a, the SHC was synthesized through an unsaturated coordination reaction between ethanolamine (EA) and CoCl_2_·6H_2_O in ethanol. After heating to evaporate the solvent, the dry SHC was obtained. Due to the facile fabrication process, the production can be easily scaled‐up. We prepared a series of samples by simply altering the molar ratios of ethanolamine to CoCl_2_ (1:4, 2:4, and 3:4). The morphology and microstructure of the resulting SHC have obvious changes with the variation of the molar ratios (Figures S1 and S2, Supporting Information). When the molar ratio is 2:4, the as‐prepared SHC presents a uniform blue film (Figure S1b, Supporting Information). Scanning electron microscopy (SEM) images reveal that the microstructure of SHC is constructed from hierarchical dendritic clusters with a diameter of several microns that resembles a cornflower (Figure 1c,d). The formation of the unique cornflower‐like structures follows a fractal process: as the solvent slowly evaporates, many initial nucleons of SHC generate. The continuous “random walk” of SHC drives it to preferentially attach to these nucleons. As a result, large scale cornflower‐like architectures with many branches self‐assemble on the surface of the substrate.^[^ ^22^ ^]^ The Fourier transform infrared (FTIR) spectra of pure EA, CoCl_2_, and three SHC samples are shown in Figure 1b. It is observed that the SHC samples show two sharp peaks at 1059 and 998 cm^−1^, corresponding to the CH_2_ and NH_2_ twisting vibration, respectively, which shift to lower frequency compared with pure EA (1076 and 1030 cm^−1^).^[^ ^23^ ^]^ The two weak peaks at 2950 and 2892 cm^−1^ are assigned to the CH_2_ stretching vibration.^[^ ^24^ ^]^ The peak at 1484 cm^−1^ is ascribed to the C–H bending vibration.^[^ ^25^ ^]^ The FTIR results confirm the successful coordination between ethanolamine and CoCl_2_, which is further verified by X‐ray photoelectron spectroscopy (XPS). As shown in Figure 1e, elements Co, O, N, C, and Cl all exist in SHC, with peaks located at around 781.0, 531.0, 401.0, 284.0, and 198.0 eV, respectively.^[^ ^26^, ^27^ ^]^

### 2.2 Moisture sorption performances of SHC

**Figure 2:**
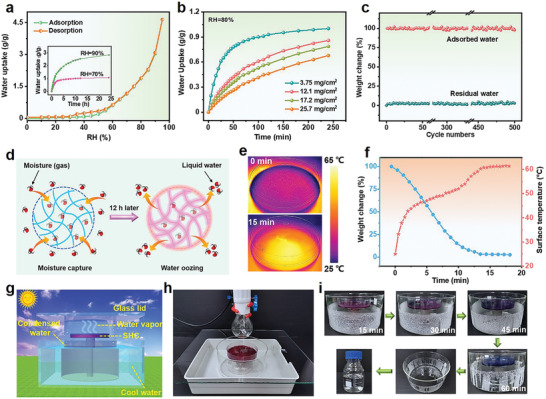
Moisture sorption behaviors of SHC. a)Water adsorption/desorption isotherms of SHC at 25 °C. Inset: Moisture adsorption kinetics of SHC at 25 °C and different RH. b) Moisture adsorption kinetics of SHC with different loading densities at 25 °C and 80% RH. c) The number of adsorption/desorption cycles. d) Schematic of the moisture adsorption and water oozing behaviors of SHC. e) Infrared images of SHC before and after exposing to one sun illumination for 15 min. f) Water release and surface temperature of SHC as a function of time under one sun illumination. g) Schematic illustration of the AWH device. h) Digital photograph of an AWH prototype. i) Digital photographs of the surface of the water collector at different times during the water collection process.

The moisture adsorption performances of the three samples were first studied at ambient air (24.5 °C, 75% RH). The saturated water uptake of the SHC prepared with the molar ratio between EA and CoCl_2_ of 2:4 is higher than other samples (Figure S3, Supporting Information), which can be ascribed to its unique cornflower‐like structure. Therefore, this sample is selected for the following experiments. The moisture adsorption/desorption behaviors of the SHC at various RH were investigated using a Vapor Sorption Analyzer from AquaLab. The obtained adsorption isotherm reveals a steep water uptake at RH above 60% (**Figure** **2**a). The saturated water uptake at RH of 60%, 70%, 80%, 90%, and 95% is measured to be 0.61, 1.15, 1.87, 3.04, and 4.64 g g^−1^, respectively. The water adsorption capacity of the SHC precedes many other hygroscopic materials, as shown in Table S1 in the Supporting Information. The SHC can quickly adsorb moisture from humid air in the first two hours (Figure 2a). Besides, the water adsorption capacity and the time required to reach saturation increase significantly with increasing the RH because more water molecules can combine with the SHC. The loading density (defined as the mass of SHC per unit area) also plays a vital role in determining the rate of water uptake. As observed in Figure 2b, the adsorption kinetics of the SHC become slower when its loading density is increased. To visually present the moisture capturing process, the dry SHC (coated on a petri dish) was placed at ambient air (25 °C, 80% RH) for a certain time and the surface of SHC was monitored via a stereomicroscope with a high‐speed camera. The SHC quickly adsorbed moisture and the color of the material gradually changed from blue to purple, and finally pink (Figure S4, Supporting Information). This can be attributed to the coordination of the adsorbed water molecules with Co (II).^[^ ^28^ ^]^ After 12 h, the surface of the SHC became blurry, indicating that the moisture adsorbed into the SHC oozed out as liquid water. This can be verified by the optical photograph, which showed that large amount of liquid water appeared in the dish (Figure S5, Supporting Information). Figure 2d demonstrates the moisture capture and water oozing behaviors of the SHC. Water molecules were adsorbed and liquefied on the surface of the SHC, and then diffused into the hierarchical dendritic networks of the SHC. The unique structure of the SHC contributed to the water storage. As a result, liquid water was directly harvested from the humid air by adsorbing moisture and oozing water using the SHC. The collected water was analyzed by inductively coupled plasma‐optical emission spectrometry (ICP‐OES) and ion chromatography. The results are summarized in Table S2 (Supporting Information), which show that the concentration of all the ions is below the WHO standards, confirming the good quality of the collected water.

The desorption of water was investigated under one sun illumination. As shown in Figure 2f, after turning on the simulated light source, water gradually evaporated. This process completed in 15 min with only ≈3% of the adsorbed water that cannot be released. The SHC achieved an obvious temperature increase due to the light absorption of the SHC (Figure S6, Supporting Information). The surface temperature of the SHC rose from 25 to 60 °C in 15 min and stabilized at this temperature (Figure 2e,f), confirming that the adsorbed water has been released. These results demonstrate the possibility of using natural sunlight or low grade heating for the regeneration of the SHC, guaranteeing the SHC an energy‐efficient and sustainable moisture sorbent. The cyclic stability of the material is another important factor to be considered for practical use. As shown in Figure 2c, five hundred regeneration cycles between adsorption at 80% RH (25 °C) and desorption upon exposure to one sun illumination (or heating at 55 °C) exhibit no obvious degradation of the adsorption capacities, demonstrating the excellent stability of the SHC.

The fast adsorption kinetics, high wateruptake, and facile regeneration enable the application of SHC in atmospheric water harvesting (AWH). Figure 2g shows the schematic illustrations of the proposed AWH device. During the moisture adsorption process, a glass petri dish (*d* = 8.7 cm) containing the dry SHC (4.0 g) was placed at ambient environment (≈85% RH) for moisture capturing until saturation. The adsorbed water was measured to be 6.5 g, corresponding to the water uptake of 1.62 g g^−1^. Then, the petri dish was placed into a sealed glass container, which was surrounded by cool water for an accelerated condensation process. The whole device was placed under one sun illumination provided by a halogen lamp for solar‐driven water release (Figure 2h). The water release‐condensation started in 15 min and finished in 60 min (Figure 2i). The total volume of water collected was 5.6 mL, realizing the production rate of 1.4 g g^−1^ h^−1^ and the water recovery rate of 86% (some water vapor escaped into the surroundings).

### 2.3 Fabrication of SHC‐Based Desiccant Dehumidifier for Simultaneous Freshwater Production and Dehumidification

**Figure 3:**
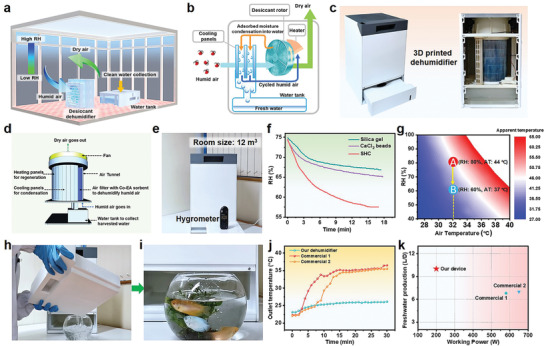
In‐house developed desiccant dehumidifier. a) Schematic illustration of continuous indoor dehumidification and water collection with the desiccant dehumidifier. b) Schematic operation of the desiccant dehumidifier. c) The prototype of the designed desiccant humidifier fabricated via 3D printing. Dimensions: 40 × 40 × 60 cm. d) Schematic inner structure of the desiccant dehumidifier. e) Dehumidifying a confined room (size: 12 m^3^, RH: ≈75%, Temperature: ≈23 °C) using the proposed desiccant dehumidifier. f) The continuous RH changes of the tested room by deploying different desiccants in the dehumidifier. The wind speed at the outlet is 1.5 m s^−1^. g) Effect of temperature and RH on apparent temperature. h) Water collected in the water tank. i) Using the collected water for raising fish. We changed the water every two days. j) The outlet temperature change of the dehumidifier over time during the dehumidification process. k) Comparative chart of working power and dehumidification volume of our desiccant dehumidifier and some commercial ones.

Extracting moisture from the air with sorbents is not only a promising strategy for freshwater production but an effective way for dehumidification. Taking the above‐mentioned advantages of the SHC, we further deployed this material in an in‐house developed desiccant dehumidifier to realize simultaneous dehumidification and freshwater production. **Figure** **3**a,b illustrates the operation mechanism of the desiccant dehumidifier. In an open indoor environment, humid air is continuously pumped into the desiccant dehumidifier and is adsorbed by the desiccant. Then dry air is transported out, realizing the function of dehumidification. At the same time, when the desiccant rotor goes through the heater, the adsorbed moisture is released and transferred to the cooling panels for condensation. Finally, the condensed liquid water is collected in the water tank, fulfilling the function of freshwater production.

A prototype of the designed desiccant dehumidifier was constructed via 3D printing (Figure 3c; Figure S7, Supporting Information). The entire assembly process is shown in the Figure S8 in the Supporting Information. Figure 3d illustrates the inner structure of the printed desiccant dehumidifier. The device consists of five key components: air filter loaded with the dry SHC for moisture adsorption, heating panels for material regeneration, cooling panels for moisture condensation, a water tank (capacity: 2L) for water collection and a fan for pumping air. The air filter is a commercial one and the loading of the sorbent is shown in the Figure S9 in the Supporting Information. The total loading amount of the dry SHC is ≈1 kg. The operation of the desiccant dehumidifier, especially the air filter, is shown in Movie S1 in the Supporting Information.

As a proof of concept, the fabricated dehumidifier was first used to dehumidify a confined room (size: 12 m^3^, RH: ≈75%, Temperature: 23 °C) (Figure 3e). A commercial hygrometer (Precision: ±2%) was utilized to monitor the RH change. Once the device is turned on, the RH of the room was observed to sharply decrease from ≈75% to 60% in 9.0 min (Figure 3f red line; Movie S2, Supporting Information). After 15 min, the RH dropped to ≈57% and tended to achieve dynamic equilibrium. The power consumption is merely 0.05 kWh (The power of the dehumidifier is 200 W). This unique dehumidification profile conforms to the recommended humidity level for a healthy and comfortable environment. For comparison, we also tested the dehumidification properties of traditionally used desiccants (Silica gel and CaCl_2_ beads) by integrating these materials into our device. As shown in Figure 3f, the RH of the room drops much slower when using silica gel or CaCl_2_ beads as desiccants. Therefore, the as‐prepared SHC could be used as a potential alternative to traditional desiccants. The reduction of RH can help improve human's thermal comfort, as it is well‐known that the RH has a paramount effect on human physiology response and thermal sensation.^[^ ^29^ ^]^ The “feel‐like” or apparent temperature (AT) can be calculated with the following equation^[^ ^30^ ^]^

(1) $$\begin{array}{ccc} AT & = & -42.379+2.04901523T+10.14333127R-0.22475541TR\\ & & -\;6.83783\times 10^{-3}T^{2}-5.481717\times 10^{-2}R^{2}+1.22874\times 10^{-3}\\ & & \times \;T^{2}R+8.5282\times 10^{-4}TR^{2}-1.99\times 10^{-6}T^{2}R^{2} \end{array}$$

where *T* is ambient dry bulb temperature (°C) and *R* is relative humidity. Table S3 (Supporting Information) lists the calculated AT under different conditions. It is obvious that the RH has significant effects on the temperature people perceived, especially at higher ambient temperatures. By using our proposed desiccant dehumidifier to reduce the RH from 80% to 60% at 32 °C, the apparent temperature can be reduced by 7 °C (Figure 3g), thus effectively improving the thermal comfort of the inhabitants. Though our device cannot reduce the temperature of the ambient, it greatly aids in mitigating the discomforts caused by high levels of RH, thereby reducing the need for air‐conditioning and cutting down energy consumption.

It is worth noting that the temperature at the outlet of our dehumidifier only shows a slight increase caused by the heat of adsorption, while the commercial ones show a significant temperature increase (Figure 3j). To examine the dehumidification capacity of the fabricated dehumidifier, the device was placed in an open environment with RH of ≈80%. After continuously working for 4 h, 1.7 L water was condensed in the water tank with (Figure 3h; Movie S3, Supporting Information). The water production rate is calculated to be 0.42 kg kg^−1^ h^−1^. The collected water can be used for raising fish (Figure 3i; Movie S3, Supporting Information). After 6 d, the fishes are still alive (Figure S10, Supporting Information), demonstrating the good quality of the collected water. The total amount of water collected in 24 h is about 10 L. To further evaluate the advantages of the fabricated desiccant dehumidifier, the dehumidification capacity and working power was compared with some commercial desiccant dehumidifiers (Figure 3k). Obviously, we have shown an energy saving of over 60% when compared to commercial desiccant‐based dehumidifiers and a higher dehumidification performance. Being deterministic of further reducing the overall energy consumption of the system, autonomous operation of the humidity management system was enabled through the integration of a precise machine‐learning assisted in‐house constructed hydrometer with the desiccant dehumidifier. The changes in electrical resistance of the SHC upon moisture sorption was utilized in the construction of the low‐cost and high precision RH sensor.

### 2.4 Humidity Sensing Properties of SHC

**Figure 4:**
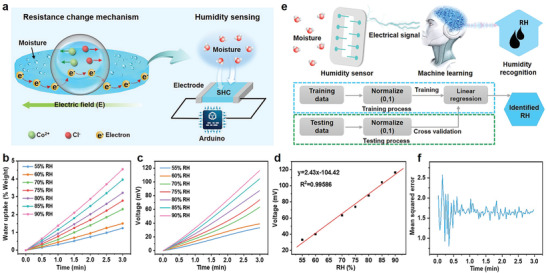
Humidity sensing performance of SHC. a) The humidity‐sensing mechanism of the SHF‐Co‐based sensor. b) The continuous water‐uptake at 25 °C and different RH levels. c) The continuous voltage changes at 25 °C upon exposure to different RH levels obtained after being processed by the Savitzky–Golay. d) The relationship between the voltage (obtained after adsorbing moisture for 3 min) and RH levels. e) Machine learning for RH prediction. f) The mean squared error (MSE) of voltage upon continuous adsorbing moisture for 3 min obtained by machine learning.

As shown in Figure S11 (Supporting Information), the resistance of the SHC (1 × 1 cm^2^) decreased sharply (from 168 to 30 kΩ) upon adsorbing moisture in 3 min, and then slowly decreased to 19 kΩ after 5 min. This is attributed to the dramatic increase of the number of transferred electrons in the adsorbed moisture as a result of the directional migration of cobalt and chloride ions in the electric field (**Figure** **4**a, left side).^[^ ^31^, ^32^ ^]^ The SHC‐based RH sensor was fabricated via transducing the resistance change to voltage change. Figure 4a (right side) illustrates the set‐up for humidity measurement with a two‐electrode configuration. The SHC is connected to Arduino NANO, which is a microcontroller and will read the voltage change continuously upon the SHC adsorbing moisture. Figure S12 (Supporting Information) shows the optical photograph of the experimental set‐up. The SHC (1 × 1 cm^2^) is spread on a glass substrate as the sensing probe.

The humidity sensing characteristics were evaluated by measuring the voltage change under different RH conditions after applying a fixed bias voltage of 3.3 V (supplied by the Arduino NANO). The response curves in Figure S13 (Supporting Information) showed the voltage increased instantly and continuously upon placing the sensor in glass boxes with a constant RH. For equal adsorption time, the voltage also increased significantly at a higher RH level. This can be attributed to the different adsorption rates. The dynamic adsorption curve in Figure 4b indicates that, as the RH increased, the rate of moisture adsorption also increases dramatically. It should be noticed that the raw data contained arbitrary noise which may undermine the sensor performance. Therefore, the raw data were processed via the Savitzky–Golay filter, which used least squares to regress a small window of data onto a polynomial and shift window by one data point iteratively.^[^ ^33^ ^]^ The processed data is shown in Figure 4c. It is observed that the data distribution roughly follows a linear relationship. Notably, the voltage response and RH also follow a linear relationship (Figure 4d). After adsorbing moisture for 3 min, the voltage increases more than 3.5 times with increasing RH from 55% to 90%.

Using the relationship between moisture adsorption and resistance change in SHC, a timesaving but accurate method to calibrate and predict the humidity level was developed by using machine learning (Figure 4e). Machine learning, a subfield of artificial intelligence, utilizes different statistical algorithms to enable computers to learn from various data types without being explicitly programmed.^[^ ^34^, ^35^ ^]^ It has been demonstrated to be an effective and sophisticated method to reveal the hidden relationship between features and labels.^[^ ^36^, ^38^ ^]^ As the data distribution roughly followed a linear relationship (Figure 4c,d), linear regression was used as the evaluation model to predict RH given past readings of voltage (Figure 4e). Finally, we computed the mean squared error (MSE) to evaluate the performance. As shown in Figure 4f, the MSE fluctuates in the first minute. This is because the distributions at different humidity levels are close to each other in the first two minutes (Figure 4c), which raises the difficulty for the regression task and results in unstable performance. After 2 min, the distributions at different humidity levels are more separate apart from each other. Therefore, the performance stays stable at around 1.6%. Overall, the results demonstrate that, by using linear regression, we can predict RH with precision of ±1.6% after collecting data of two minutes or more.

### 2.5 Demonstration of Autonomous Humidity Control System

**Figure 5:**
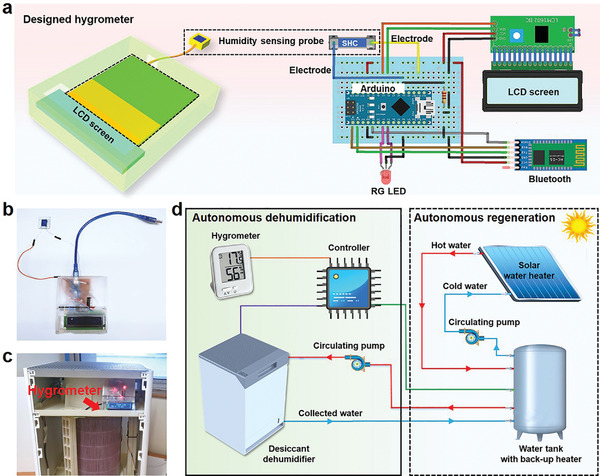
Design and operation of autonomous dehumidification/regeneration system. a) Schematic illustration of the configuration of the proposed hygrometer. b) The prototype of the proposed hygrometer. c,d) An autonomous dehumidification/regeneration concept driven by renewable solar energy. The right part is the dehumidification process, and the left part is the regeneration part. The collected water during dehumidification process can be used for the cycling water.

The humidity sensing ability of the SHC was deployed to fabricate a hygrometer for providing data to the fabricated dehumidifier for autonomous operation. **Figure** **5**a shows the configuration of the proposed hygrometer. In this device, the SHC functions as a potentiometer and is connected to the 3.3‐volt power supply pin and the A1 pin on Arduino NANO (Figure S14, Supporting Information). Arduino NANO, as a microcontroller, will read the voltage change continuously upon the SHF‐Co adsorbing moisture. Then Arduino NANO will convert the voltage to RH and send it to the LCD screen. For operation convenience, the readings of RH can be sent to an android phone via Bluetooth. Besides, an RG led is connected to Arduino NANO via pin D5, D6 to monitor the connection status of the Bluetooth. When the Bluetooth is online, the RG led will turn to green color, otherwise, it shows a red color. Figure 5b shows the prototype of the proposed hygrometer. To evaluate the feasibility of the hygrometer, we use it to monitor the RH of a given space and compare the results with the ones obtained from commercial hygrometers (Figure S15 and Table S4, Supporting Information). The RH detected by our hygrometer is close to the ones detected by the commercial humidity sensor, especially number 1 that has high precision of ±2% (price: US$365), demonstrating its feasibility in real applications. Figure S16 (Supporting Information) displays the sending of recorded RH to an android phone via the Bluetooth. It should be emphasized that our device costs less than US$5 but has high precision, which is superior to many commercial hygrometers (Table S4, Supporting Information).

The fabricated hygrometer was integrated with the dehumidifier to construct an autonomous humidity control system, as shown in Figure 5c. The Arduino NANO, as a microcontroller, enables the communication between the hygrometer and the dehumidifier which enables autonomous operation (Figure 5d left part). Once the reading of the hygrometer reaches the highest RH setpoint the dehumidifier will be switched on automatically and start to dehumidify the room. When the RH reading reaches the lowest RH setpoint, the dehumidifier will automatically shut down, turning into the power saving mode. Movie S4 (Supporting Information) shows the autonomous operation of the integrated system with the highest and lowest RH setpoint of 75% and 63%, respectively. Since the fabricated dehumidifier also has the ability for water collection, we further propose an autonomous regeneration process for the desiccant material. As shown in Figure 5d (right part), the regeneration system consists of a solar water heater, a water tank with a back‐up heater, a circulating pump. The water collected by the dehumidifier can be used for the cycled water. This system is also connected to the controller. When the dehumidifier turns into the power saving mode, the regeneration process for the desiccant will start autonomously. Normally, in sunny weather, the solar water heater receives solar radiation to heat the water used for regenerating desiccants. This process is particularly suitable for tropical regions and countries. During cloudy weather when the solar radiation is insufficient, required necessary thermal energy to run the system will be provided by the back‐up heater integrated with the water tank.

## 3 Conclusion

In summary, we have designed and constructed a highly efficient, low‐energy, and autonomous desiccant‐based humidity control system that can realize dehumidification and atmospheric water harvesting simultaneously. The system comprises of an in‐house fabricated desiccant dehumidifier and hygrometer (sensor), both of which are fabricated with the as‐prepared SHC. The SHC has superior moisture sorption properties with fast sorption rate, high water uptake and easy regeneration, and shows a dramatic change in electrical resistance upon capturing moisture. The dehumidifier can dehumidify a confined room from RH of 75% to 60% in 15 min with energy consumption of merely 0.05 kWh, saving more than 60% of energy compared with the commercially available desiccant‐based dehumidifiers. Moreover, the reduction in RH from 80% to 60% at 32 °C can results in an apparent cooling by 7 °C, thus effectively improving the thermal comfort of the inhabitants. By utilizing the adsorbed moisture, we have also demonstrated the possibility of simultaneous freshwater harvesting with a water yield of about 10 L in 24 h. Machine learning assisted in‐house developed low cost and high precision hygrometers enabled the autonomous operation of the humidity management system enabling additional energy savings. This work sheds light on the development of a new generation of low‐energy and high‐efficiency desiccant‐based humidity control systems with an additional capability of sustainable water production.

## Conflict of Interest

The authors declare no conflict of interest.

## Supporting information

Supporting Information

Supplemental Movie 1

Supplemental Movie 2

Supplemental Movie 3

Supplemental Movie 4
