# Supplementary material for: Machine‐Learning‐Assisted Autonomous Humidity Management System Based on Solar‐Regenerated Super Hygroscopic Complex
Source: Adv Sci (Weinh). 2021 Feb 1;8(6):2003939. doi: 10.1002/advs.202003939 (PMC7967090; doi:10.1002/advs.202003939)
Supplement: Supplementary file 1 — Supporting Information [file ADVS-8-2003939-s004.pdf]

© 2020 Wiley-VCH GmbH

ADVANCED SCIENCE
Open Access

Supporting Information

for *Adv. Sci.*, DOI: 10.1002/advs.202003939

Machine learning-assisted autonomous humidity management system based on solar-regenerated super hygroscopic complex

Xueping Zhang, Jiachen Yang, Hao Qu, Zhi Gen Yu,
*Dilip Krishna Nandakumar, Yaoxin Zhang, Swee Ching Tan**

Supporting Information

Machine learning-assisted autonomous humidity management system based on solar-regenerated super hygroscopic complex

*Xueping Zhang, Jiachen Yang, Hao Qu, Zhi Gen Yu, Dilip Krishna Nandakumar, Yaoxin Zhang, Swee Ching Tan**

Methods

Synthesis of SHC. Firstly, 0.95 g of $CoCl_2 \cdot 6H_2O$ was dissolved in 5 mL of ethanol, followed by adding of certain amount of ethanolamine (60, 120 or 180 μL) under stirring, which led to the formation of a blue complex. Finally, the liquid products were dried at 70 ºC for about 15 min to evaporate the solvent.

Water adsorption/desorption experiments. The water adsorption/desorption isotherm was measured at 25 ºC on an AquaLab Vapor Sorption Analyzer. Before the experiment, 300 μL of the liquid sample was added into the glass container and dried in a vacuum oven to form a uniform thin film. For the water adsorption experiment conducted at ambient air, the liquid sample was added into a petri dish and dried in a vacuum oven, as shown in Figure S1. The solar-driven water desorption experiment was performed with a solar simulator (Newport 92250A-1000, AM1.5) with standard 1 sun illumination. Weight change of the SHC was tracked by a properly calibrated electronic balance (Sartorius CP224S). The infrared images of the top surface of the SHC were taken by a thermal imaging camera (FLIR One Pro). The temperature readings in related measurements were obtained from the infrared images.

Fabrication of atmospheric water harvesting (AWH) device. A petri dish covered with the SHC was used for atmospheric moisture capture. The petri dish was placed in a glass container, which was sealed by a glass lid for water condensation and collection. A water tank containing cool water was used to condense the water vapor.

Fabrication of desiccant dehumidifier. All the supporting parts and the outer covers of the dehumidifier was prepared through 3D printing. The 3D printer we used is Up Box+, and the material we used is ABS. The assembly process was shown in Figure S8.

Humidity sensing tests. The room-temperature humidity sensing properties of the SHC-based humidity sensor were characterized using a two-electrode configuration. Figure S12 shows the measurement setup. A glass substrate coated with the SHC (1×1 cm^2) served as the sensing probe, which is connected to Arduino NANO. The MegunoLink was utilized to

monitor the voltage change after a fixed bias voltage of 3.3 V (supplied by the Arduino NANO) was applied to the sensor. In each measurement, the sensor was exposed to RH for 3 min, followed by heating at 55 ºC for 5 min.

Machine learning. The dataset contained 7 instances and each instance consisted of two parts: past readings of voltage and the corresponding RH. We trained the linear regression model using 6 instances (i.e., past readings of voltage as the training feature and corresponding RH as the training label) and used the trained linear regression model to predict RH on the remaining test instance. Then we compared the predicted RH to its true value. The above evaluation procedures were repeated 7 times so that every instance can be predicted as a test instance (such evaluation procedures are also known as leave-one-out cross-validation).

Characterization. Scanning electron microscopy (SEM) experiments were carried out on a Zeiss Supra 40VP scanning electron microscope operated at 10 kV. X-ray diffraction (XRD) data were measured using a D8 ADVANCE (Bruker, Germany) X-ray diffractometer with Cu Kα radiation (λ = 1.5406 Å). X-ray photoelectron spectra (XPS) were obtained from an ESCALABMKII X-ray photoelectron spectrometer with an excitation source of Al Ka radiation. The UV-Vis spectra were recorded on a Shimadzu 1800 UV-Vis spectrophotometer. Fourier transform infrared (FT-IR) spectra were recorded using an Agilent CARY 660 FT-IR spectrophotometer. The light absorption spectra were measured by an Agilent Cary 7000 UV-Vis-NIR spectrometer with an integrating sphere.

Movie S1: The operation of the fabricated dehumidifier.

Movie S2: Dehumidifying a confined room with the fabricated dehumidifier.

Movie S3: Water collected from the dehumidifier for raising fish.

Movie S4: The autonomous operation of the dehumidifer enabled by integrating the fabricated hygrometer.

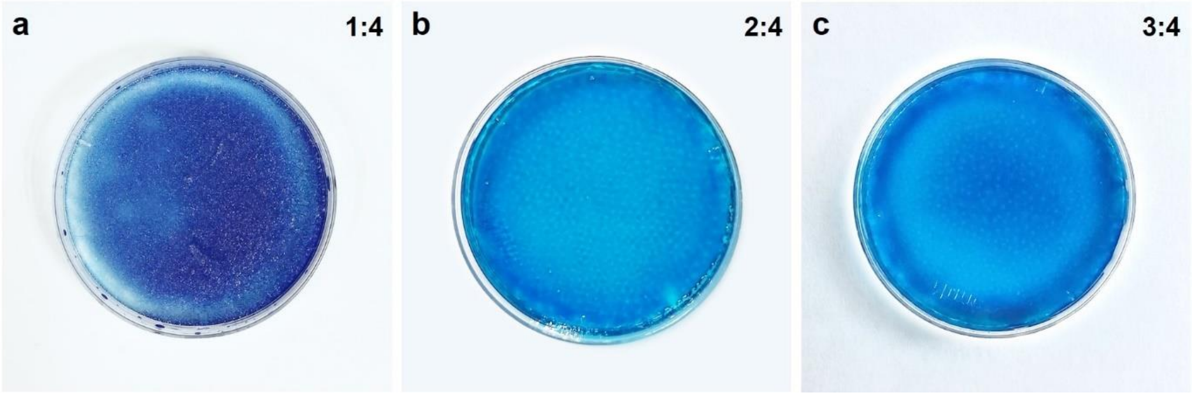

Figure S1. The morphology of the dehydrated SHC prepared with different molar ratios of ethanolamine to $CoCl_2$.

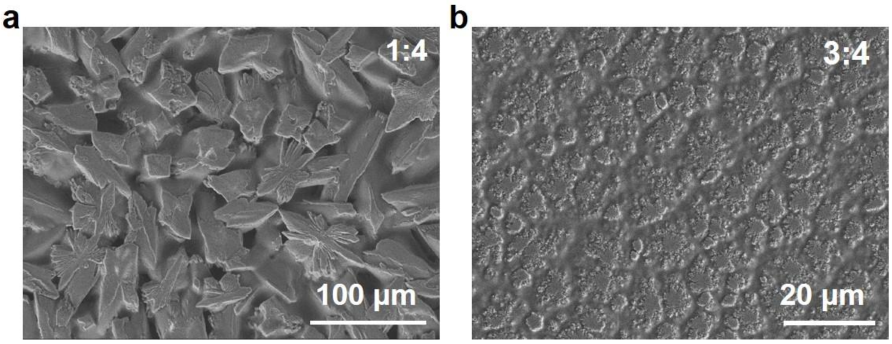

Figure S2. SEM images of SHC prepared with molar ratios of ethanolamine to $CoCl_2$ of 1:4 and 3:4, respectively.

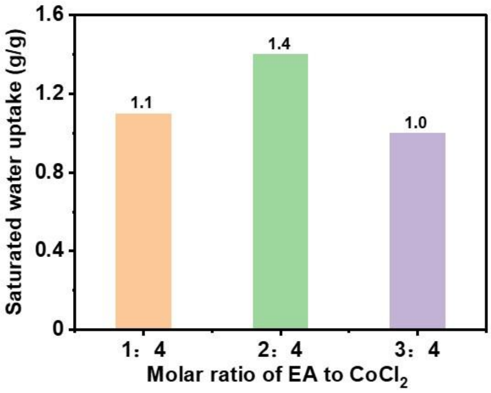

Figure S3. The saturated water uptake of different samples.

Table S1 Comparison of the maximum water uptake and the water desorption temperature of some hygroscopic materials

	Water uptake (g g^{-1}) at 90% RH	Regeneration temperature (℃)
Our Co-complex	3.0	60
Our Cu-complex[1]	2.0	60
Our Zn-complex[2]	2.3	60
MOF-801[3]	0.4	70
MIL-101 (Cr)[4]	1.7	90
Silica gel[4]	0.3	120

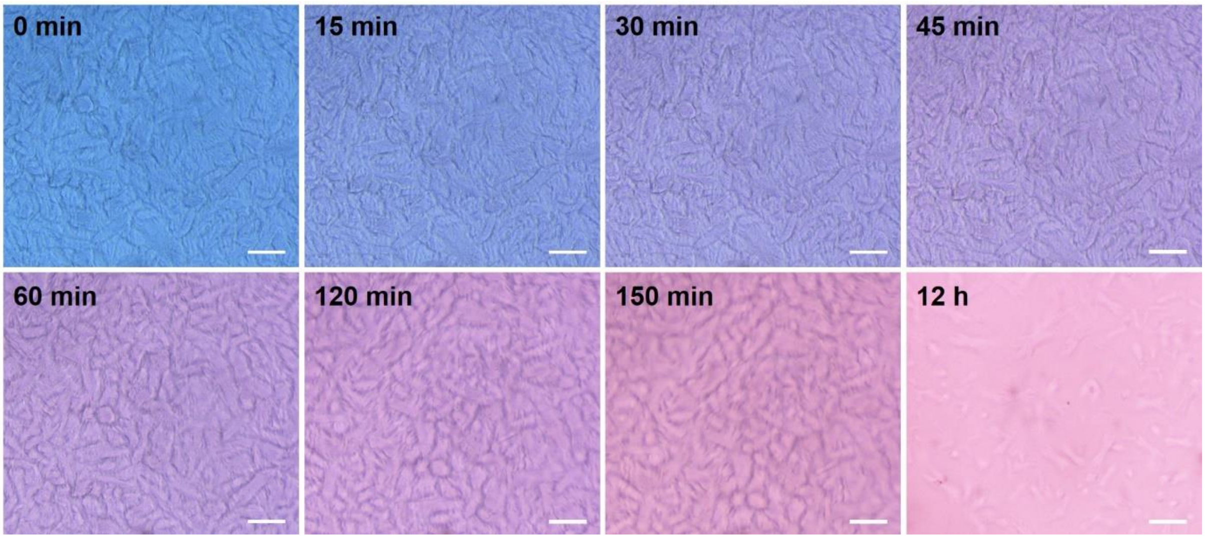

Figure S4. The real processes of color change recorded by a stereomicroscope with a high-speed camera. The scale bar is 20 μm.

It is observed that the SHC has interesting color change, from blue to purple, and finally pink, upon exposure to moisture.

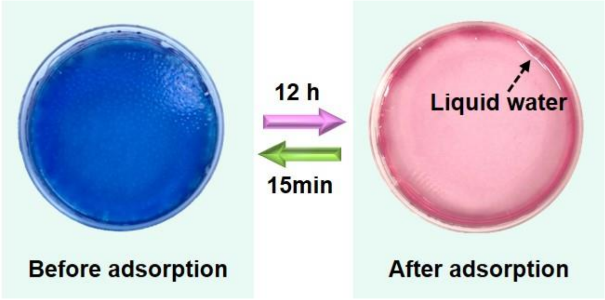

Figure S5. Optical photographs showing water oozing and color change of SHC complex at 25 °C and 80% RH.

Table S2 Comparison of quality of water collected from AWG with the WHO's drinking water standards.

	Determined values (ppm)	WHO standards (ppm) [5]
Co	1.2 ppb	2 ppb
Cl	29.7	250
Br	0.33	ND
F	ND	1.5
SO_4^{2-}	0.27	250
$NO3^-$	0.38	50

ND: Not determined

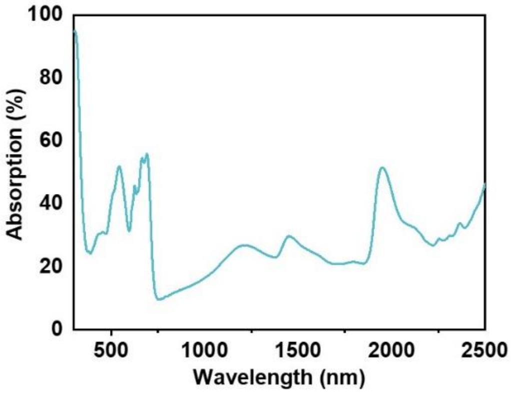

Figure S6. Light absorption spectra of the SHC in the full solar spectrum range (250-2500 nm).

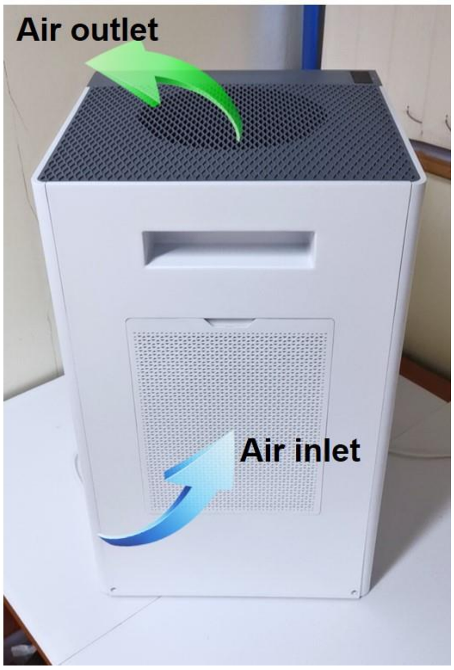

Figure S7. The back of the 3D printed desiccant dehumidifier.

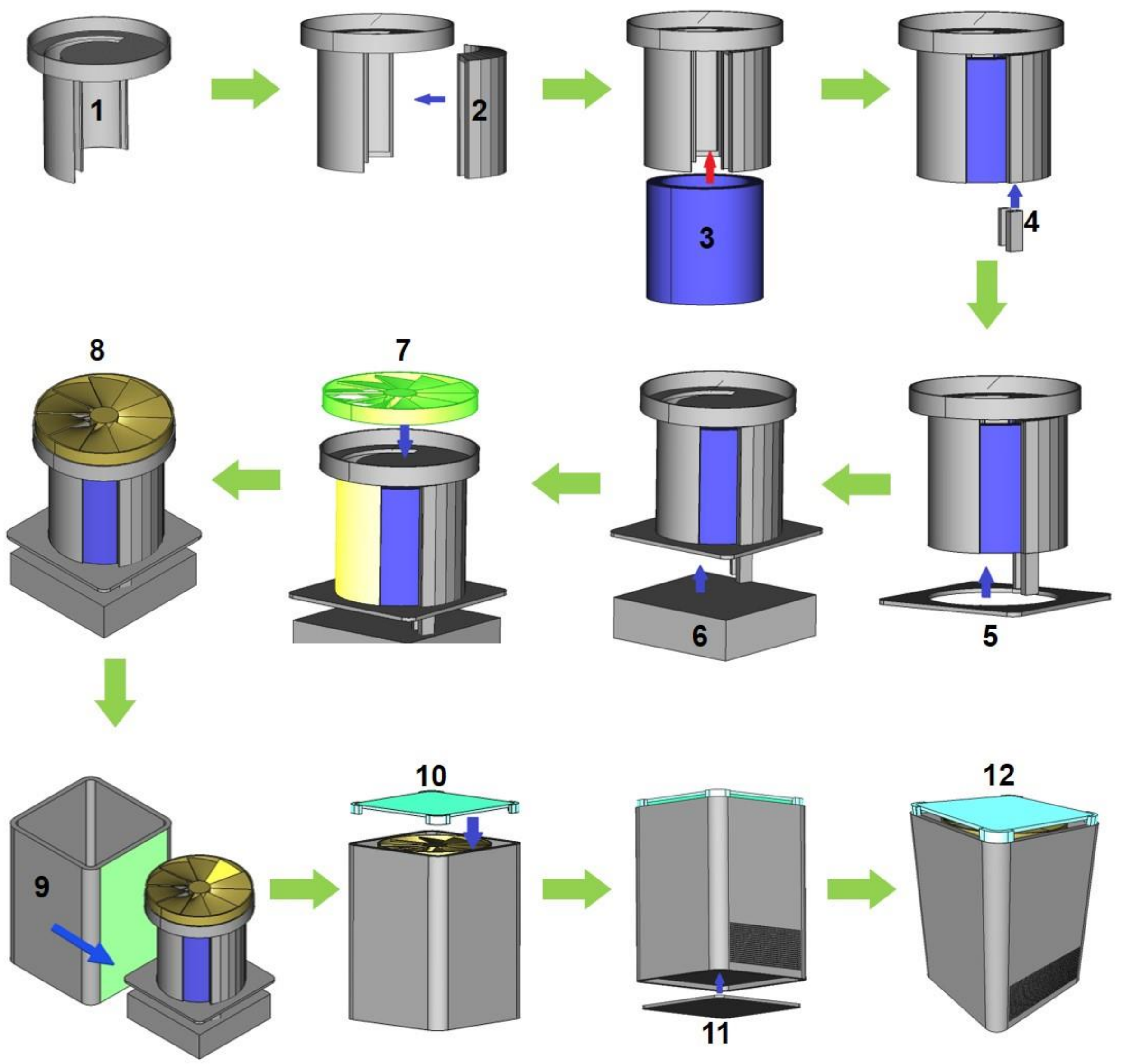

Figure S8. The assembly process of the desiccant dehumidifier. All the supporting parts and the outer covers are obtained via 3D printing. (1) Fan hold and air tunnel, (2) Heating and cooling panels, (3) Air filter loaded with SHC, (4) Water pipe, (5) Base, (6) Water tank, (7) Fan, (8) Overview, (9) Case, (10) Top cover, (11) Bottom cover, (12) Overview.

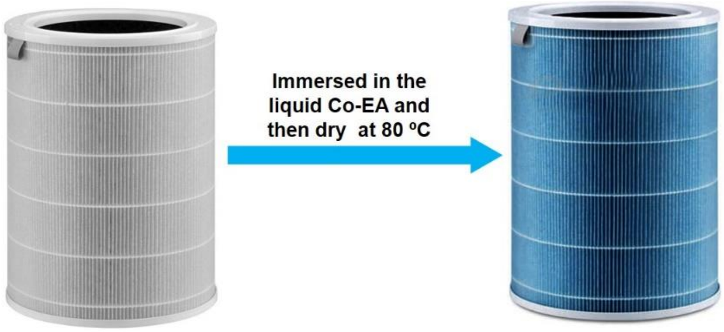

Figure S9. The loading process of the SHC in a commercial air filter.

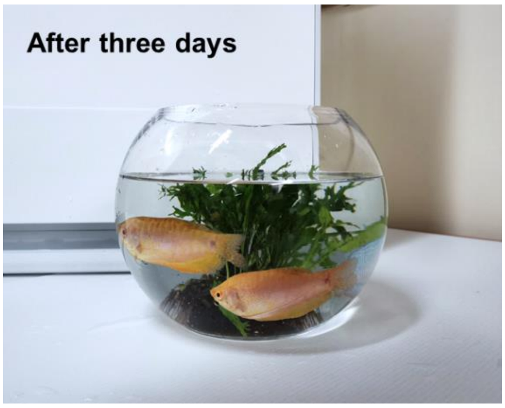

Figure S10. Photograph of the fishes raised in the collected water for three days.

Table S3. Effect of temperature and relative humidity on apparent temperature.

Relative humidity (%)	Air temperature (℃)													
	27	28	29	30	31	32	33	34	35	36	37	38	39	40
40	27	28	29	30	31	32	34	35	37	39	41	43	46	48
45	27	28	29	30	32	33	35	38	39	41	43	46	49	51
50	27	28	30	31	33	34	36	38	41	43	46	49	52	55
55	28	29	20	32	34	36	38	40	43	46	49	52	55	59
60	28	29	31	33	35	37	40	42	45	48	51	55	59	63
65	28	30	32	34	37	39	41	44	48	51	55	59	63	67
70	29	31	33	35	38	40	44	47	50	54	58	63	67	72
75	29	31	34	36	39	42	46	49	53	58	62	67	72	77
80	30	32	35	38	41	44	48	52	57	61	66	71	77	83
85	30	33	36	39	43	47	51	55	60	65	70	76	82	88
90	31	34	37	41	45	49	54	58	64	69	75	81	88	95
95	32	35	39	43	47	52	57	62	68	74	80	87	94	101
100	33	36	40	44	49	54	60	66	72	78	85	92	100	104

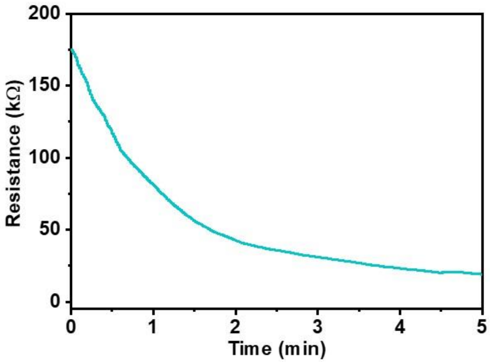

Figure S11. The continuous changes of the resistance of SHC upon exposure to ambient air (25 ºC, 80% RH).

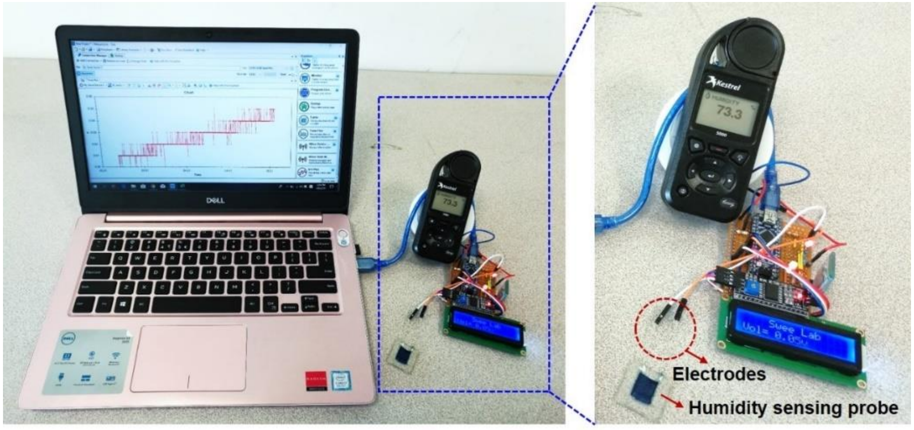

Figure S12. The recording of voltage changes with the proposed humidity sensing device upon exposure to a certain RH.

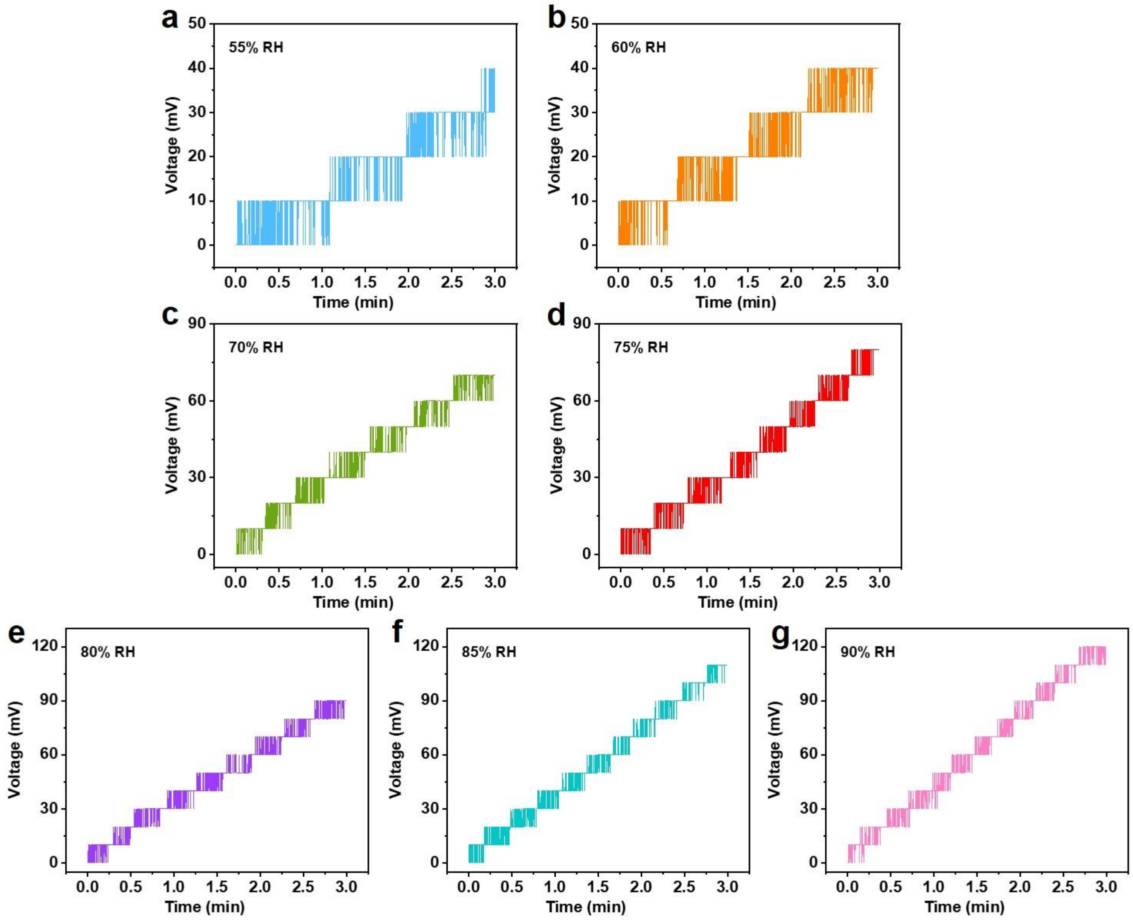

Figure S13. The continuous voltage changes at 25 ºC upon exposure to different RH levels.

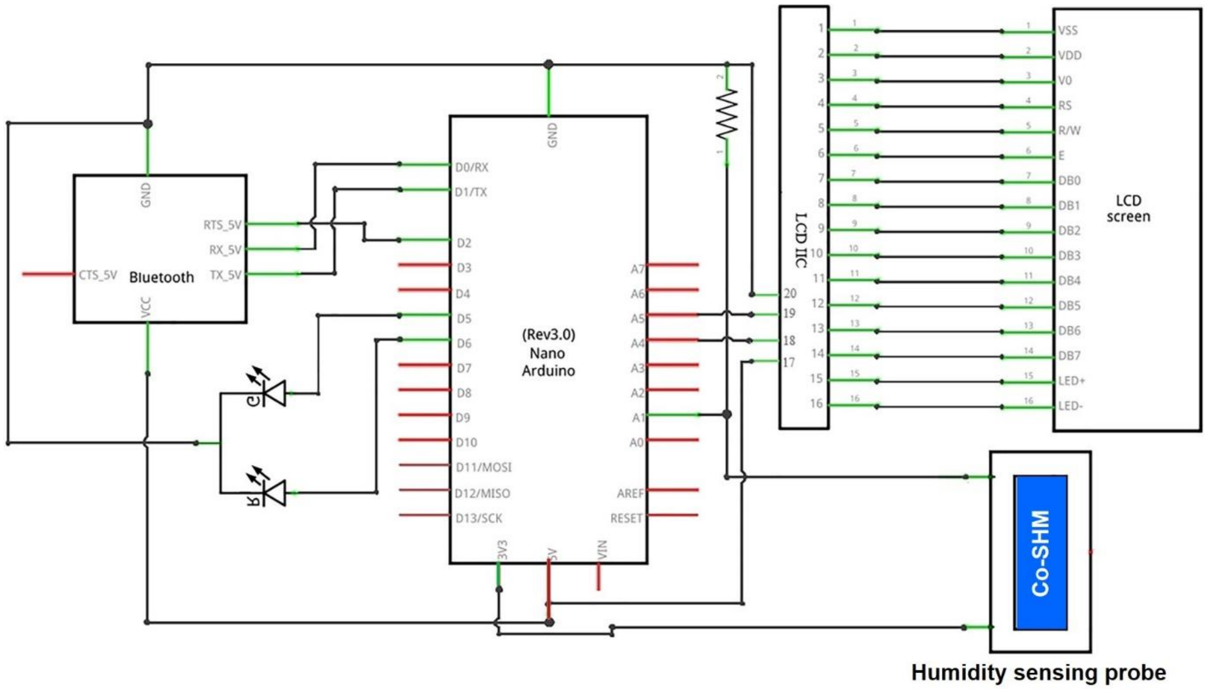

Figure S14. The circuit diagram of the hygrometer.

In this device, the SHC is coated on a glass channel and connected with the 3.3volt power supply pin and the A1 pin on Arduino NANO. The SHC-based sensing probe can function as a potentiometer. Arduino NANO, as a microcontroller, will read the voltage change continuously. Upon exposure to different RH, the resistance of the SHC is different, that means by measuring the different voltage, we would get a certain RH. The Arduino NANO will convert the voltage to RH reading and send it to the LCD screen. The LCD screen is connected to Arduino NANO with the A4 and A5 pin via an IIC board. Meantime, the RH and voltage readings will be sent to an android phone via Bluetooth which is connected to Arduino NANO via the D0, D1, and D2 pin. If Bluetooth is connected to any android phone, it will send a message to Arduino NANO via pin D2. An RG led is connected to Arduino NANO via the D5, D6 pin. When a message has been received by Arduino NANO saying that Bluetooth is online, the RG led will turn to green color. Otherwise, it will show a red colour.

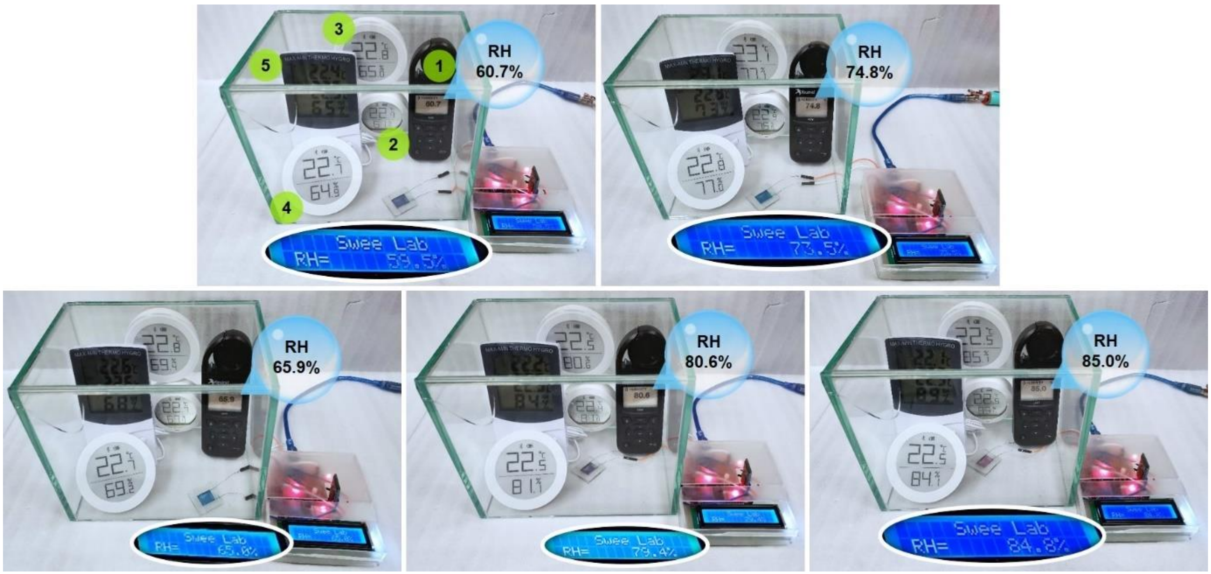

Figure S15. Monitoring the RH with the proposed humidity sensor and some commercial ones.

Table S4. Comparison of the RH monitored by our proposed hygrometer and commercial ones.

	RH	RH	RH	RH	RH	Precision	Cost
**Our hygrometer**	**59.5**	**65.0**	**73.5**	**79.4**	**84.8**	**±1.6%**	**US$5.0**
Commercial 1	60.7	65.9	74.8	80.6	85.0	±2%	US$365
Commercial 2	61.9	67.4	76.5	81.0	85.0	±10%	US$45
Commercial 3	65.0	69.4	77.1	80.6	85.1	±10%	US$43
Commercial 4	64.9	69.2	77.0	81.0	84.1	±10%	US$43
Commercial 5	65	68	73	84	89	±10%	US$14

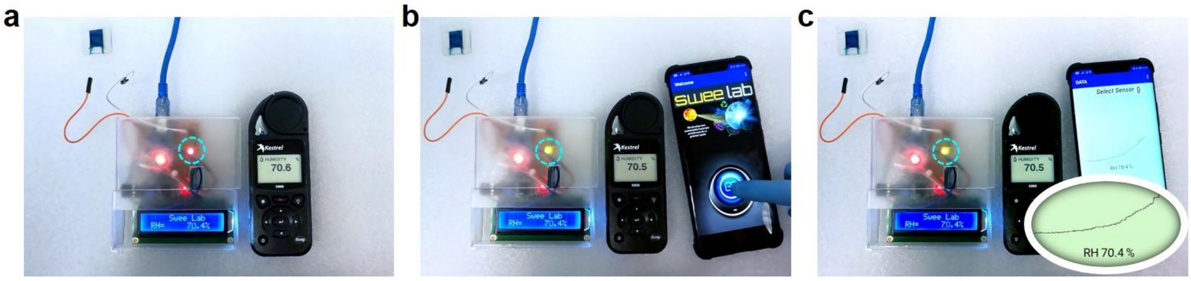

Figure S16. Demonstration of the connection of the hygrometer to an android phone via Bluetooth.

References

[1] J. Yang, X. Zhang, H. Qu, Z. G. Yu, Y. Zhang, T. J. Eey, Y. W. Zhang and S. C. Tan, *Adv. Mater.*, **2020**, 2002936.

[2] D. K. Nandakumar, S. K. Ravi, Y. X. Zhang, N. Guo, C. Zhang, S. C. Tan, *Energy Environ. Sci.*, **2018**, *11*, 2179-2187.

[3] H. Kim, S. Yang, S.R. Rao, S. Narayanan, E. A. Kapustin, H. Furukawa, A.S. Umans, O. M. Yaghi, E. N. Wang, *Science*, **2017**, *356*, 430-434.

[4] Y.-K. Seo, J. W. Yoon, J. S. Lee, Y. K. Hwang, C.-H. Jun, J.-S. Chang, S. Wuttke, P. Bazin, A. Vimont, M. Daturi, S. Bourrelly, P. L. Llewellyn, P. Horcajada, C. Serre, G. Férey, *Adv. Mater.* **2012,** *24*, 806-810.

[5] 2017 WHO Guidelines for Drinking Water Quality: First Addendum to the Fourth Edition, Vol. 109, American Water Works Association 2017.
